# Microfluidic fabrication of microcarriers with sequential delivery of VEGF and BMP-2 for bone regeneration

**DOI:** 10.1038/s41598-020-68221-w

**Published:** 2020-07-16

**Authors:** Erfan Dashtimoghadam, Farahnaz Fahimipour, Nikita Tongas, Lobat Tayebi

**Affiliations:** 1 Marquette University School of Dentistry, Milwaukee, WI USA; 2 Adams School of Dentistry, University of North Carolina at Chapel Hill, Chapel Hill, NC USA

**Keywords:** Engineering, Materials science

## Abstract

Wound instability and poor functional vascularization in bone tissue engineering lead to lack of tissue integration and ultimate failure of engineered grafts. In order to harness the regenerative potential of growth factors and stimulate bone healing, present study aims to design multifunctional cell therapy microcarriers with the capability of sequential delivery of essential growth factors, bone morphogenetic protein 2 (BMP-2) and vascular endothelial growth factor (VEGF). An on-chip double emulsion method was implemented to generate monodisperse VEGF encapsulated microcarriers. Bio-inspired poly(3,4-dihydroxyphenethylamine) (PDA) was then functionalized to the microcarriers surface for BMP-2 conjugation. The microcarriers were seeded with mesenchymal stem cells (MSCs) using a dynamic culture technique for cells expansion. Finally, the microcarriers were incorporated into an injectable alginate-RGD hydrogel laden with endothelial cells (ECs) for further analysis. The DNA and calcium content, as well as ALP activity of the construct were analyzed. The confocal fluorescent microscopy was employed to monitor the MSCs and tunneling structure of ECs. Eventually, the capability of developed microcarriers for bone tissue formation was examined in vivo. Microfluidic platform generated monodisperse VEGF-loaded PLGA microcarriers with size-dependent release patterns. Microcarriers generated with the on-chip technique showed more sustained VEGF release profiles compared to the conventional bulk mixing method. The PDA functionalization of microcarriers surface not only provided immobilization of BMP-2 with prolonged bioavailability, but also enhanced the attachment and proliferation of MSCs. Dynamic culturing of microcarriers showcased their great potential to boost MSCs population required for stem cell therapy of bone defects. ALP activity and calcium content analysis of MSCs-laden microcarriers loaded into injectable hydrogels revealed their capability of tunneling formation, vascular cell growth and osteogenic differentiation. The in vivo histology and real-time polymerase chain reaction analysis revealed that transplantation of MSC-laden microcarriers supports ectopic bone formation in the rat model. The presented approach to design bioactive microcarriers offer sustained sequential delivery of bone ECM chemical cues and offer an ideal stabilized 3D microenvironment for patient-specific cell therapy applications. The proposed methodology is readily expandable to integrate other cells and cytokines in a tuned spatiotemporal manner for personalized regenerative medicine.

## Introduction

Bone healing is a multifaceted developmental process which involves multiple signaling molecules in a specific localization and orchestration to induce vascularization and osteogenesis^1–3^. Vascularization plays a leading role in tissue repair and regeneration^4,5^. However, conventional constructs for bone tissue engineering are poorly vascularized, which typically leads to their weak integration and survival^6^. On the other hand, slow penetration of the host vasculature in the use of tissue replacements in critical-sized defects usually causes necrosis within the central region of the engineered tissues^7,8^.

Autologous transplant of healthy bone tissue is the current bone graft gold standard^9,10^. However, the size of the defect hinders vascularity of a non-vascularized bone graft^9,11–13^. Moreover, radiation therapy, scar tissues and systemic diseases—creating poor vascularity—make grafting procedures much less predictable and often necessitate free flap transfers^14–17^.

Cell therapy-based regenerative approaches that graft autologous or non-autologous cells have been proposed to overcome the limitation of available clinical bone grafts^18^. However, the long-term survival and functional state of the cells to actively proliferate and differentiate still require further adjustments. Manipulation of the extracellular matrix (ECM) microenvironment to mimic the cell niche is a promising approach to enhance stem cell based clinical regenerative outcomes^19,20^. The niche highly dynamic key components comprise direct interactions between stem cells and secreted signaling molecules—ECM physical and chemical cues, along with environmental changes such as hypoxia^19–21^.

The biomaterial-based delivery systems as an adjunctive strategy to regenerative medicine approaches have been presented to utilize the potential application of stem cells^22,23^. In this combinatorial strategy, different signaling molecules—including growth factors—are delivered locally at the site of defect. Yet, the key to harness the reparative potential of signaling molecules is to design a multifunctional system to tune the release rate and duration of multiple growth factors to mimic that of natural bone healing. Hence, an ideal stem cell carrier for bone regeneration should mimic natural microenvironment cues to recruit biologics, induce vascularization and stimulate stem cell expansion and differentiation toward osteogenic and vasculogenic lineages^19,24,25^. In this view, ECM mimetic carriers promise an ideal approach through harvesting the advantages of microparticle-mediated cellular therapy^19^.

In bone repair, bone marrow-derived mesenchymal stem cells (MSCs) are an extensively used cell source for implementation in regenerative craniofacial applications^26,27^. Herein, we aim to advance expansion and differentiation of MSCs through resembling structures in nature to design bioactive microcarrier that can accomplish remarkable feats to serve the unmet bone regeneration need. The designed capability of microcarriers provides cells anchorage, combined with vascular endothelial growth factor (VEGF) and bone morphogenetic protein (BMP-2) sequential delivery to enhance viability, proliferation and fate of the transplanting cells.

Although a variety of vehicles such as microparticles have been investigated for growth factor delivery, a robust predictive method for clinical application has yet to be achieved^28–30^. The conventional bulk mixing approaches—which are typically employed to fabricate delivery vehicles for growth factors—have several drawbacks including polydispersity, unpredictable spatial release profile, limited bioactivity, and low encapsulation efficiency. These shortcomings ultimately prevent eventual translation of such particulate systems for clinical applications^24,31^. To this end, biomanufacturing of fine-tuned carriers promises several advantages such as uniform size and loading efficiency, which can provide homogeneous release throughout the delivery region^32–35^. The microfluidic platform has shown capability to generate monodisperse microcarriers with precise encapsulation of bioactive agents to finely tune the spatiotemporal bioavailability of loaded cargo^32–35^. Uniform microcarriers for bone cell therapy ensure localized accessibility of transplanted cells to growth factors over the predetermined regeneration time span^36–38^. To the best of our knowledge, current study is the first report on microfluidic-assisted polymeric microcarriers for cell therapy applications.

**Figure 1:**
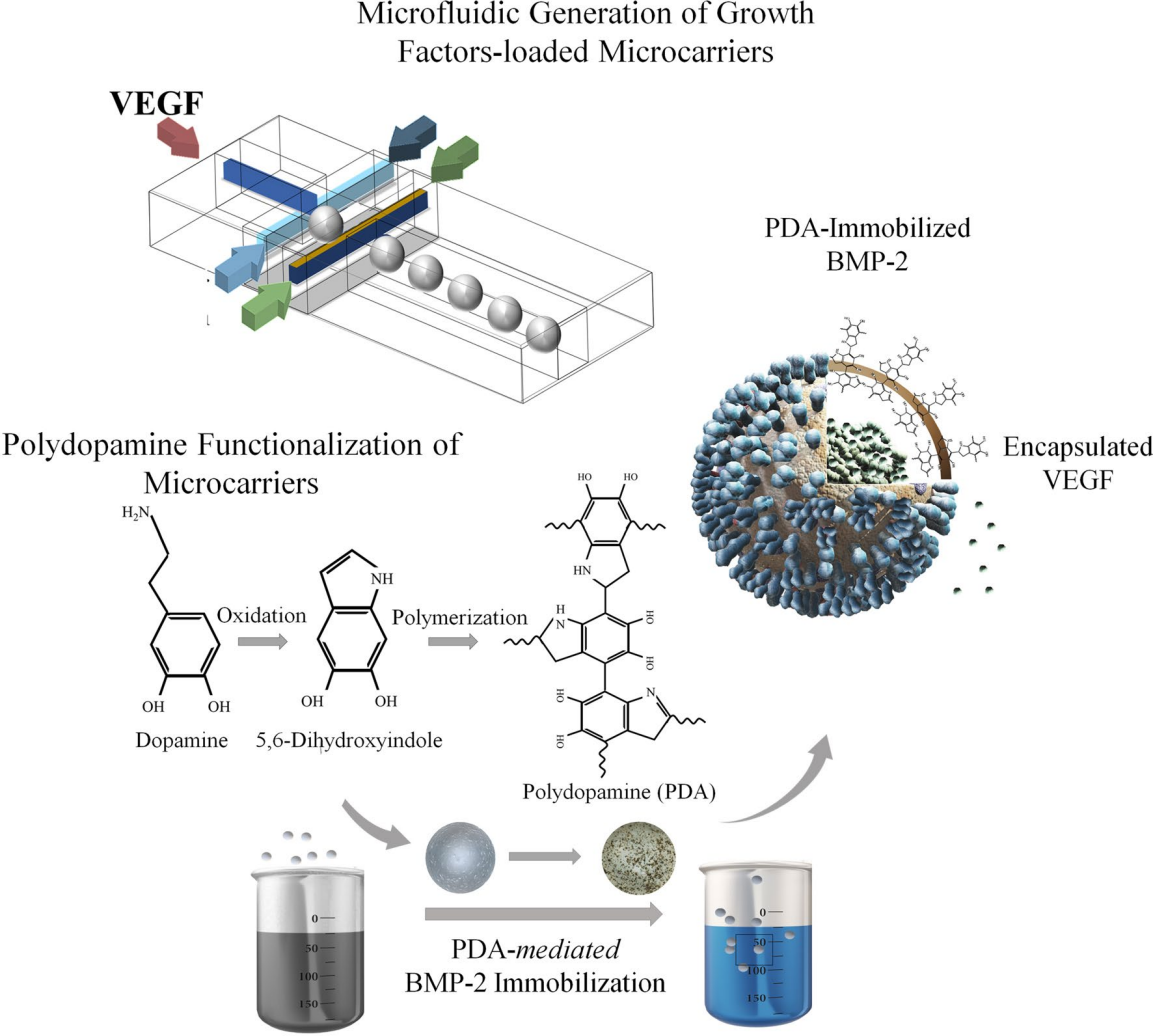
The schematic representation of the developed approach to synthesis the multifunctional microcarriers with the capability of sequential delivery of bone morphogenetic protein 2 (BMP-2) and vascular endothelial growth factor (VEGF).

Herein, a microfluidic fabrication technique is implemented to generate monodisperse VEGF-loaded PLGA microcarriers. The microcarriers are then functionalized through a bio-inspired approach by poly(3,4-dihydroxyphenethylamine) (PDA) to not only immobilize BMP-2 with prolonged bioavailability, but also to enhance biomineralization and cellular adhesion (Fig. 1). It should be mentioned that the generated functional microcarriers are usable to extensively expand and guide the mesenchymal stem cells through dynamic culturing techniques for bone stem cell therapy.

## Material and methods

### Microfluidic fabrication of VEGF-loaded microcarriers

VEGF was encapsulated into PLGA microcarriers though a microfluidic-assisted double emulsion solvent evaporation technique. The inner flow was prepared through dissolution of 3 μg VEGF-165 (Cells Applications, Inc.) in 0.2 ml sterile phosphate buffer saline (PBS), and the middle carrier stream composed of poly (lactic-co-glycolic acid) (PLGA 50:50) was dissolved in dichloromethane at 3 or 10w/v% polymer concentration. The outer stabilizing flow was 1 *w/v*% aqueous solution of poly (vinyl alcohol) (PVA, Sigma-Aldrich) to produce a water–oil–water (W/O/W) double emulsion.

**Figure 2:**
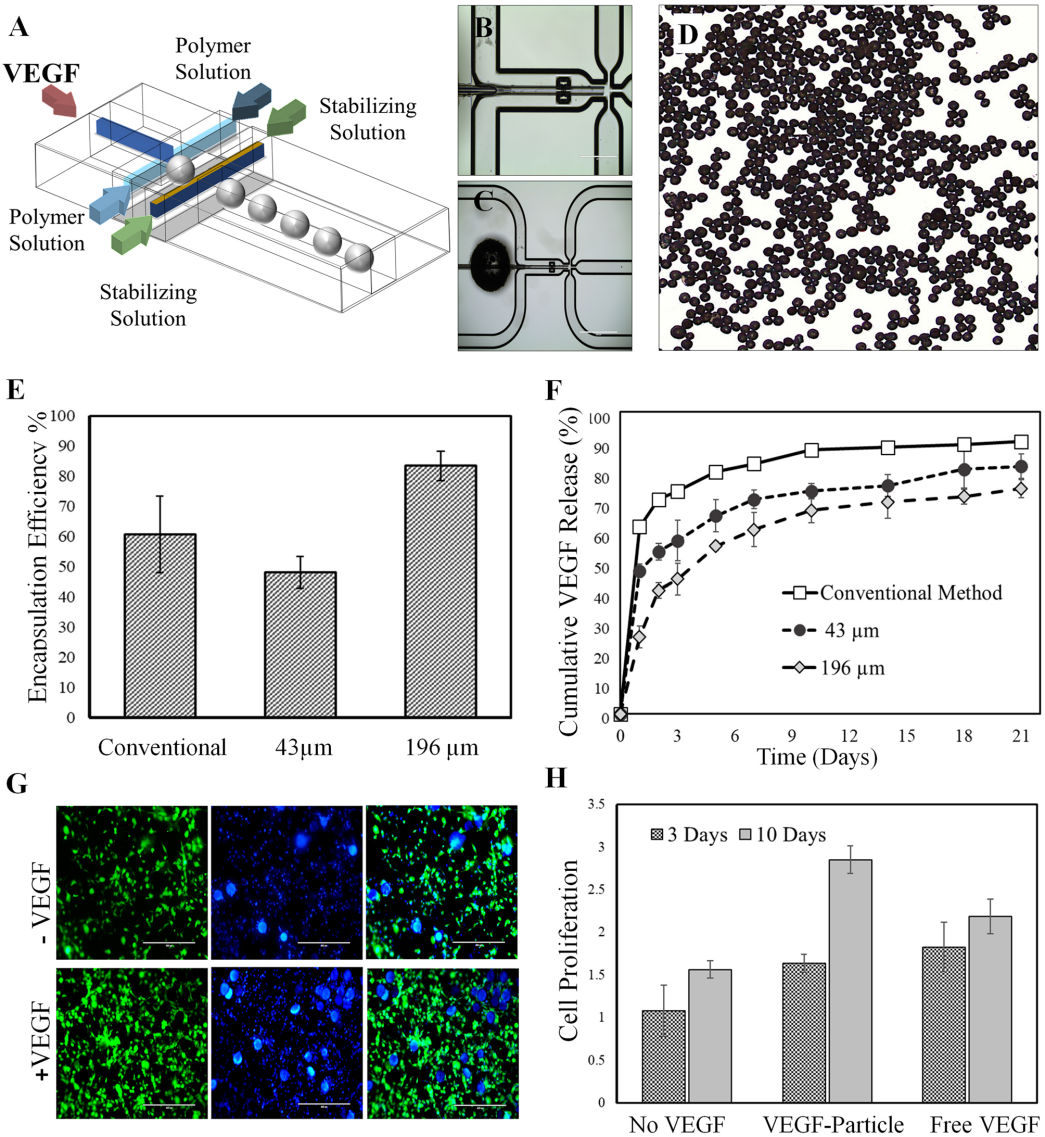
(**A**,**B**) The schematic representation of the Microfluidic based method to fabricate polymeric microcarriers. (**D**) The microfluidic synthesized microcarriers are spherical and monodisperse in diameters. (**E**) The encapsulation efficiency and (**F**) release pattern of VEGF from various microcarriers over 3 weeks. (**G**) The fluorescent images of the 3D-cultured HUVECs with or without VEGF after 10 days. (**H**) The viability of HUVECs after 3 days, and 10 days cultured with VEGF loaded microcarriers, free VEGF and control.

Microfluidic-assisted growth factor encapsulation was conducted on a chip with five inlets including one for growth factor solution as the inner flow, two for polymer solution as middle flows and two for stabilizing solution as the outer flows (Fig. 2A). Emulsion phases of the W/O/W emulsion were injected into the chip using syringe pumps (Chemyx, Inc.). PTFE tubing (Cole Parmer) was used to connect syringes to the inlets of the chip. Various compositions with different flow rates were assessed. The inner, middle, and outer fluid phases were injected at the flow rate of 1, 5, and 70 μL/min, respectively.

Similar phase solutions were used to prepare VEGF-loaded microparticles using conventional bulk mixing method. The first emulsion was obtained through vertexing the PLGA and VEGF solutions mixture. The resultant W/O emulsion was then added dropwise into the stabilizing solution stirring at 800 rpm to obtain a W/O/W double emulsion. Finally, the emulsion was incubated at room temperature to allow gradual evaporation of DCM.

### Kinetics of VEGF release

The entrapment efficiency of VEGF was calculated based on 1 mg VEGF-incorporated microcarriers after degradation in 1 mL 0.05 N NaOH while stirring for 24 h. Afterward, the solution was centrifuged at 5,000 rpm and the supernatant was analyzed for VEGF content by ELISA kit (Cell Applications Inc.) as described elsewhere^24^. In order to study VEGF release profile, loaded microcarriers were suspended in 1 mL PBS at pH 7.4, and incubated at 37 °C. At scheduled time intervals of 1, 2, 3, 5, 7, 10, 14, and 21 days following incubation time, a release sample was collected and replaced with the same volume of fresh filtered medium. The content of VEGF was recorded as nanogram per milliliter of solution.

### VEGF bioactivity and HUVECs proliferation

The bioactivity of VEGF released from microcarriers was determined by analyzing the proliferation of Human umbilical vein endothelial cells (HUVECs, Cell Application, USA) in vitro. HUVECs were cultured in modified endothelial cell growth medium (EGM-2). The EGM-2 was modified (mEGM-2) for the experiments by removing the supplied VEGF from the culture medium. Experiments were completed with HUVEC cells from the fourth passage. The cells proliferation was quantified by seeding the HUVECs on a 24-wells culture plate, either VEGF-loaded or unloaded microcarriers were added. PrestoBlue (Invitrogen) cell vitality assay was conducted following supplier’s protocol at predetermined post-culture time points. The fluorescence intensity (ex: 560 nm and Em: 590 nm) was analyzed via a microplate reader (Synergy HTX, BioTek). All experiments were implemented at least in triplicate. Further, HUVECs were also monitored by fluorescent microscopy using CytoPainter ER Staining Kit, Green Fluorescence (Abcam).

### BMP-2 immobilization on microcarriers

Bone morphogenetic protein 2 (BMP-2) was immobilized onto microcarriers through their surface treatment using poly(3,4-dihydroxyphenethylamine) (PDA). Microcarriers were immersed in 2 mg/mL 3,4-dihydroxyphenethylamine hydrochloride (Sigma-Aldrich) dissolved in a 10 mM Tris buffer for 6 h with mild shaking. The microcarriers were then separated and washed with deionized water. Subsequently, functionalized microcarriers were incubated in 500 ng/mL BMP-2 solution at 37 °C under mild shaking overnight. Microcarriers were then washed with sterile PBS, freeze-dried and stored in a sterile condition for further usage. The immobilization efficiency of BMP-2 was measured based on washout BMP-2 content by an ELISA kit (Cell Applications Inc.) as described elsewhere^19^. The content of BMP-2 was recorded as ng/ml of solution. In order to study BMP-2 release profile, the conjugated microcarriers were suspended in 1 mL PBS at pH 7.4, and incubated at 37 °C. At scheduled time during 28-days following incubation time, a release sample was collected and replaced with the same volume of fresh filtered medium.

### Dynamic culture of microcarriers for Mesenchymal stem cells expansion

Mesenchymal stem cells (MSCs) were expanded using a dynamic culture technique. Spinner flasks were first treated with Sigmacote (Sigma-Aldrich) according to the manufacturer’s instruction. Microcarriers were then incubated with 5,000 cells cm^2^ at static condition for 1 h at 37 °C, 95% humidity and 5% CO_2_. Afterward, the cell culture suspension was dynamically agitated constantly at the rate of 30 rpm. The 50% of cell culture medium was exchanged every other day.

The expanded MSCs on the microcarriers were collected according to a protocol reported elsewhere^39,40^. In order to harvest cells, the agitation was stopped and culture medium was removed from the spinner flask. Microcarriers were then washed twice with PBS. Afterward, cells were detached by using TrypLE Express (Thermo Fisher Scientific). The cells were isolated from the microcarriers by filtration using a sterile 75 μm filter (Millipore). Cells were finally harvested by centrifuge and re‐suspended in the culture medium.

### DNA quantification assay

DNA quantification assay was conducted at 3, 7 and 14 days. The DNA quantification was carried out by means of the Quant-iT PicoGreen dsDNA Assay Kit (Thermo Fisher Scientific) following the manufacturer’s instruction. In brief, samples were first digested in 1 ml of 4 M guanidine hydrochloride buffer (pH = 7.5). Afterward, 50 μl of DNA standards were incubated with 150 μl of 1 × PicoGreen reagents. Finally, fluorescence measurements (Ex 480 nm/Em 520 nm) were performed by a spectrophotometric microplate reader (Synergy HTX, BioTek).

### Fabrication of transplantable cell-laden microcarriers with sequential delivery of VEGF and BMP-2

VEGF-loaded PLGA microcarriers were generated using a microfluidic platform, followed by their surface functionalization with PDA to conjugate BMP-2 as described above. In the next step, microcarriers were inoculated with 20,000 cells cm^2^. The inoculation was done at static condition for 1 h at 37 °C, 95% humidity and 5% CO_2_ followed by overnight dynamically agitation at 30 rpm. The MSCs loaded microcarriers were then encapsulated in a HUVECs-laden injectable hydrogel.

To prepare HUVECs-laden hydrogel, alginate-RGD (NovaMatrix) solution was mixed with 1 × 10^6^ HUVECs. Then, microcarriers cultured with MSCs were mixed with the gel. Subsequently, the gel formulation was mixed with calcium sulfate slurry, injected into a cell insert (Corning) for solidification and incubated at 37 °C. The solidified hydrogels were transferred to the cell culture medium, which was replaced every 2 days. The morphology of HUVECs was monitored by fluorescent microscopy. The cells encapsulated through hydrogel were fixed with 4% paraformaldehyde for 20 min, then permeabilized with 0.1% Triton X-100 in PBS for 10 min. For actin staining, cells were incubated with 1 × Green Fluorescent Phalloidin Conjugate working solution using F-actin Staining Kit (Cytopainter, ab112125, abcam, USA) for 1 h. Finally, samples were gently rinsed with PBS to remove excess dye prior to fluorescence microscopy (EVOS FL Auto Cell Imaging System, ThermoFisher Scientific).

The hydrogels were investigated for alkaline phosphatase (ALP) activity and total calcium content using o-cresolphthalein colorimetric assay. At day 7, 14 and 21, samples were crushed on ice into 5 volumes of Assay Buffer, and centrifuged at 4 °C at top speed for 15 min to remove insoluble materials. The supernatant was collected and stored at − 80 °C. ALP activity was assessed in triplicates by the ALP assay kit (Abcam, USA), according to the manufacturer’s instruction. In brief, 50-μl samples were diluted with 50 μl ALP buffer (0.1 M glycine, 1 mM MgCl2, 1 mM ZnCl2, pH 10.4), and then incubated with 100 μl of 5 mM p-nitrophenyl phosphate (p-NPP) at 25 °C for 60 min in darkness. A stop solution terminated the reaction, and the absorbance of p-nitrophenol was recorded at 405 nm by means of a microplate reader (Synergy HTX, BioTek). The ALP activity was normalized to the total DNA content.

### Surgical procedure

All surgical procedures were implemented according to an experimental protocol approved by the Institutional Animal Care and Use Committee (IACUC) of Marquette University^20^. All experiments were performed in accordance with relevant guidelines and regulations. Male Fischer 344 rats aged 7–8 weeks were housed in a temperature and humidity-controlled environment on a 12/12 h light/dark cycle, with food and water made available ad libitum. 5 × 10^6^ MSCs seeded on microcarriers were encapsulated in alginate-RGD (NovaMatrix) solution before transplantation. Four experimental groups were examined: (i) PDA-treated microcarriers (as control group), (ii) VEGF-encapsulated microcarriers, (iii) BMP‐2 conjugated microcarriers, and (iv) VEGF-encapsulated microcarriers conjugated with BMP-2. All microcarriers were pre-treated using PDA functionalization. The cell-laden constructs were subcutaneously implanted into the dorsum of rats. Transplantation was performed on five animals for each group, two transplants in each. The implanted constructs were collected six weeks post-implantation. For histological analysis, collected tissue samples were fixed in 4% paraformaldehyde for 24 h and then decalcified in 10% ethylenediaminetetraacetic acid solution at 4 °C for 6 weeks. Samples were dehydrated in increasing concentrations of ethanol and embedded in paraffin. 5 µm sections cut using a microtome, then deparaffinized and rehydrated in a graded descending ethanol series (100%, 90%, and 70% ethanol, 5 min each; dH_2_O for 10 min) before being stained with hematoxylin and eosin (H & E). Tissue samples were pulverized to perform qPCR assay (*n* = 4). The total RNA was extracted using Trizol reagent (Invitrogen), following the instructions provided. RNA (1 µg) was used for reverse transcription into complementary DNA (cDNA) (SuperScript VILO cDNA Synthesis Kit, Invitrogen). For the quantitative real-time PCR (qPCR), aliquots of synthesized cDNA were added to with SYBR Green PCR Master Mix (Applied Biosystems, USA) and cycled. Primers for PCR were as follows: (1) Runt-related transcription factor 2 (RUNX2) fwd: 5´-TCTTCACAAATCCTCCCC-3´rev: 5´-TGGATTAAAAGGACTTGG-3´, (2) Osteocalcin (OCN) fwd: 5´- CATGAGAGCCCTCACA-3´rev: 5´-AGAGCGACACCCTAGAC-3´, (3) vascular endothelial growth factor (Vegfα) fwd: 5´- ACTCATCAGCCAGGGAGTCT -3´rev: 5´-GGGAGTGAAGGAGCAACCTC -3´, and (4) *β*-ACTIN fwd: 5´-AGCCATGTACGTTGCTA-3´ rev: 5´-AGTCCGCCTAGAAGCA-3, which was used as a reference gene.

### Statistical analysis

All results were reported as mean ± standard deviation (SD), then statistically analyzed by one-way ANOVA. A *p*-value < 0.05 was considered to be statistically significant.

## Results and discussion

Lack of tissue vascularization is recognized as one of the main challenges in applying bone tissue engineering approaches for clinical applications. Deficient functional vascularization in bone grafting leads to a lack of graft integration, and the ultimate failure of engineered bone. In fact, cell viability in the deep regions of large defects is compromised as a result of poor vascularization, followed by hypoxia and inadequate nutrient transport^9,11–13^. Although use of microvascular free flaps can enhance clinical outcome, but drawbacks such as significant donor site morbidity, long operation time and high cost make it a high-risk procedure^41^. In line with this evidence, VEGF inhibition has been found to disturb bone restoration in experimental models^42,43^.

Osteogenic and vasculogenic growth factors are the key players during bone repair. Hence, to mimic the natural bone repair, growth factors need to be delivered in a similar spatiotemporal fashion to effectively differentiate cells toward osteogenic and vasculogenic lineages^44^. Embedding VEGF along with BMP-2 within engineering tissue constructs is an effective approach to promote the osteogenesis and vascularization process. Hence, the key to harness their reparative potential is to design a multifunctional system to tune the release time and duration of VEGF and BMP-2 to mimic that of natural bone healing^45–50^.

VEGF release must be highly localized, otherwise its excess dose can promote tumor growth rate in off-target areas. Besides, VEGF release must be controllably sustained. The uncontrolled release of VEGF cause disordered development of blood vessels, malignant tumor angiogenesis and assembly of leaky vessels^51,52^. VEGF is responsible for early bone fracture healing and will promote endochondral and intramembranous ossification. There is a temporal communication between blood vessels and bone to maintain the integrity of skeletal system. However, bolus injection of VEGF can induce inappropriate neovascularization in unwanted sites and enhance the risk of tumor growth^53^. On the other hand, due to the short half-life, the new tissue is usually transient and disappears over time. The newly formed blood vessels require sustained and controlled VEGF signaling to stabilize^51–53^.

In order to achieve high entrapment efficiency and controllable spatiotemporal release of VEGF, this work presents microfluidic encapsulation of VEGF into PLGA microcarriers for cell transplantation applications. Despite excellent biocompatibility approved by Food and Drug Administration (FDA) which makes PLGA an attractive polymer for clinical applications, lacking functional moieties for cell adhesion and biomineralization limits its potential for bone tissue engineering^54,55^. Thus, various techniques such as coating with inorganic particles (*e.g.*, hydroxyapatite) and covalent or non-covalent grafting of biomolecules can be carried out to modify the inert polymer surface^54,55^. Inspired by marine organisms to induce wet adhesion, poly(3,4-dihydroxyphenethylamine) (PDA) was coated onto the PLGA microcarriers in the present work. The PDA coating inspired by mussel adhesion is formed by spontaneous oxidation and self-polymerization of dopamine under alkaline conditions. The formation of a robust nanoscale layer of PDA is the result of combined pathways of non-covalent self-assembly. It has been known that the self-assembly of dopamine and 5,6-dihydroxyindole and polymerization of the indole leads to bioactive strong adhesive layer of PDA^27,49^.

The microfluidic platform utilized to implement W/O/W double emulsion method to encapsulate VEGF in PLGA microcarriers is displayed in Fig. 2A–C. The microfluidic chip is consisted of five inlets: one for VEGF aqueous solution as the inner flow, two for PLGA solution in organic solvent as middle flows, and two for stabilizing solution as the outer streams. As displayed in Fig. 2D, the fluidic generated microcarriers are spherical and monodisperse. By changing the polymer concentration in the middle flow, VEGF-loaded microcarriers with two different diameters of 43 ± 6 and 196 ± 14 μm were generated. Entrapment efficiency (EE) is of great importance for delivery systems. EE for microfluidic generated microcarriers was found to be 82 ± 7%, while that of for conventional particles were obtained as 67 ± 16% (Fig. 2E). The superior EE in the case of microfluidic carriers is attributed to the dropwise growth factor encapsulation at microscale, along with retained bioactivity of growth factor through alleviated mixing condition and minimized organic solvent contact. Figure 2F shows the in vitro release profile of VEGF from PDA-coated PLGA microcarriers. As seen, microcarriers demonstrate a biphasic release pattern; an initial burst effect which is due to the release of growth factor trapped in superficial regions of microcarriers, and prolonged release in the second stage due to the slow diffusion of the growth factor from the microcarriers matrix^24,54^.

The release of biomacromolecules from biodegradable polymeric microcarriers occurs through a combination of different mechanisms^55–59^. Typically, it occurs through desorption of surface-bound molecules (predominantly exists in the case of particulate systems produced through bulk mixing methods), diffusion through the polymer matrix and erosion of the polymeric carriers^24,56^. It should be noted that microfluidic generated microcarriers showed more sustained release than corresponding microcarriers synthesized by the conventional bulk double emulsion solvent evaporation method. Such observation can be attributed to the capability of the on-chip technique to more localize bioactive molecules in the inner regions of the PLGA microcarriers, which results in less surface-bound molecules, and extend their release time. The release pattern of the microfluidic generated microcarriers was found to be size-dependent^54–57^. For a given diffusion rate, the VEGF efflux per mass increases as the particle size decreases, which is due to the increase of surface area to volume ratio. Besides, quicker water penetration into the smaller particles and faster degradation of such particles due to higher volumetric surface area enhance the release rate^57–59^.

Figure 2G shows the fluorescent images of the 3D cultured human umbilical vein endothelial cells (HUVECs) in the presence of VEGF-loaded and unloaded particles after 10 days. Proliferation assay shows that the microcarriers containing VEGF have significantly increased the proliferation rate of HUVECs after 3 and 10 days compared to both bulk microcarriers and free VEGF (*p* < 0.05). It can be inferred that VEGF encapsulated PLGA microcarriers can preserve its biological activity in vitro (Fig. 2H).

**Figure 3:**
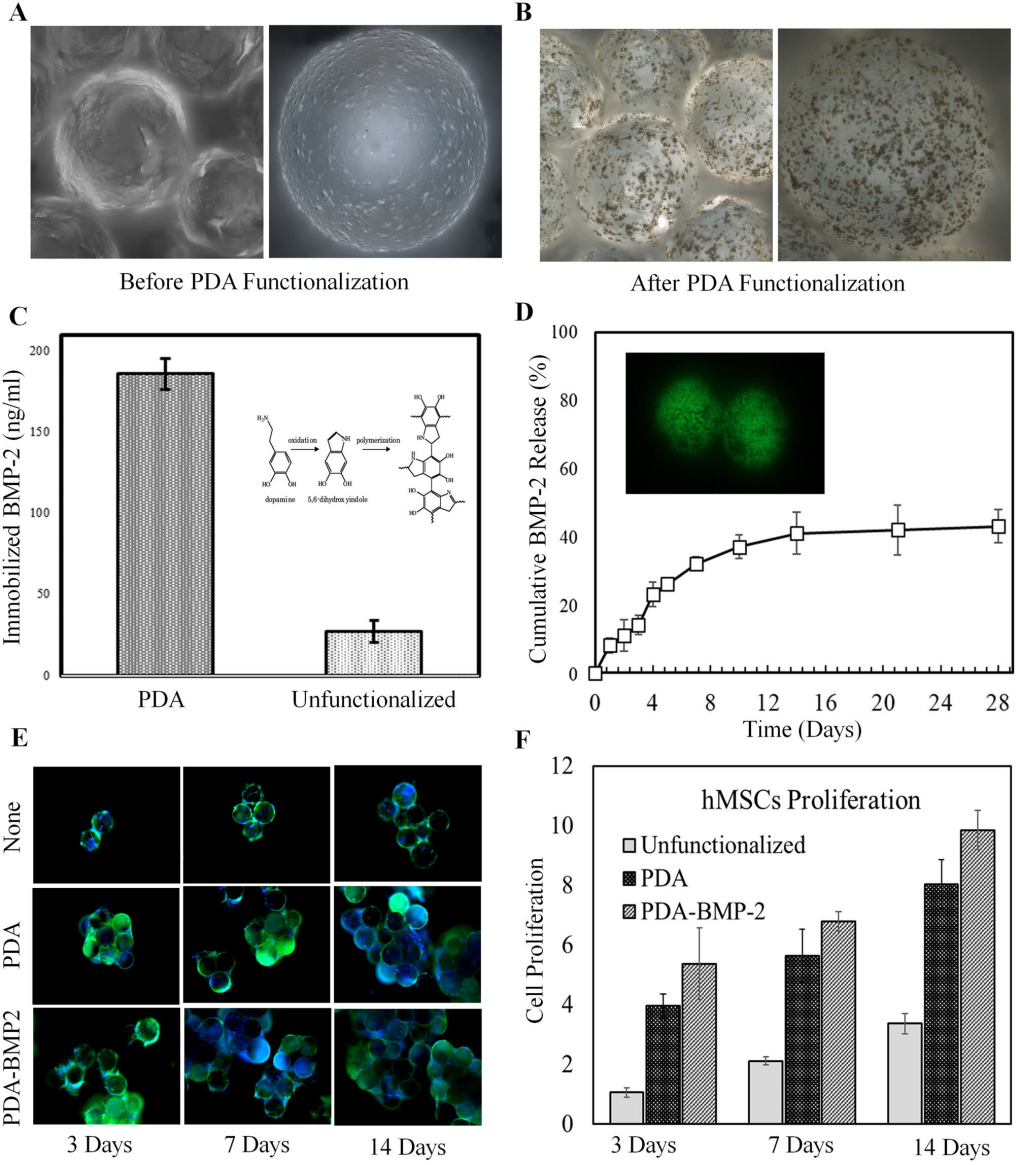
(**A**,**B**) Mussel-inspired poly(3,4-dihydroxyphenethylamine) functionalization on polymeric microcarriers. (**C**) BMP-2 immobilization efficiency of PDA functionalized and unfunctionalized microcarriers. (**D**) The release pattern of BMP-2 over PDA functionalized microcarriers in 4 weeks. (**E**) The fluorescent images of the MSCs attached on the microcarriers over 14 days of dynamic culturing. (**F**) Mesenchymal stem cells (MSCs) proliferation on unfunctionalized, PDA and PDA-BMP-2 microcarriers over 14 days of dynamic culturing.

The fluidic-generated microcarriers were functionalized using PDA treatment to achieve immobilization of BMP-2, enhance biomineralization, and increase cellular adhesion. Figure 3A,B show the 3D laser scanning images of microcarriers before and after PDA functionalization, respectively. Surface modification of microcarriers using PDA forms a stable coating that is adherent to the polymer surface. Such post-modification and functionalized layer allow for the immobilization of bioactive molecules via Michael addition and/or imine formation. It has been shown that polyester scaffolds modified with PDA chemistry facilitate cells proliferation and migration^60–63^.

Several researches have shown that MSCs in the presence of BMP-2 more effectively induce bone formation than MSCs alone in both ectopic and orthotopic sites^19,64^. Among BMPs, *recombinant human* BMP-2 is extensively used as a potent osteogenic factor in craniomaxillofacial reconstruction, like alveolar ridge augmentation; however, poor control over its burst release causes excess load of BMP in bone graft, and consequently increases the clinical cost^65–67^. In native ECM, BMP-2 exists in soluble as well as matrix-bound forms, where appropriate binding of signaling molecules to the ECM induce prolonged activation of receptors and differential phosphorylation. The significant advantage of growth factor immobilization is enhanced efficiency, or in other terms, a small amount and controlled presentation of the molecule^68–70^. Figure 3C displays PDA functionalization, which has significantly increased BMP-2 immobilization to 185 ± 9 ng/ml in comparison with 27 ± 6 ng/ml for the unfunctionalized microcarriers. It is found that PDA functionalization has increased BMP-2 immobilization efficiency onto surface from 12.4 ± 2.76% to 85 ± 4.1%. Further, the sustained release profile in vitro shown in Fig. 3D confirms the efficient immobilization of BMP-2 onto microcarriers surface.

In the native bone healing process following an inflammatory stage, blood vessels and MSCs are recruited to the defect area and proliferate. In the next phase, MSCs differentiate to osteoblasts. MSCs delivery for bone regeneration is being actively explored for the bone injuries and disorder treatment^60^. It is well understood that to achieve a successful cell therapy for bone regeneration, scale-up and expansion of MSCs is critical. To this end, increasing the number MSCs along with regulating their fate is critical to meet the clinical needs^39,71–73^. In other words, successful cell therapies mainly rely on providing adequate cell numbers required for regenerative outcome, so the need to expand the therapeutic cells becomes “mission critical”^18^.

Clinical cell therapies for regenerative medicine require development of a defined culture for large expansion and differentiation before transplantation. There are two main methods of 3D cell culture, to use cell containing particles or utilizing suspended microcarriers as adherent surfaces^74–76^. A critical advantage of microcarriers is their high surface area to volume ratio, so the number of passage and the complications associated with delivery of necessary supplements are alleviated^75–77^. It has been shown that microcarriers support MSC expansion and long-term self-renewal, and capable to provide the clinically relevant cell numbers. Microcarriers permit expansion and direct differentiation of MSCs within a 3D microenvironment. It has been reported that MSCs expansion can be reached as high as 20-fold per passage using microcarriers. Additionally, microcarrier- or aggregate-based cultures offer improved access to the nutrients and signaling molecules^74^.

The proliferation of MSCs cultured onto microcarriers comprising BMP-2 using the dynamic technique was compared with the functionalized microcarriers without BMP-2 and bare microcarriers (Fig. 3E). The live/dead staining images present the viability and proliferation of MSCs over 3, 7 and 14 days. As shown in Fig. 3F, functionalization of polymeric microcarriers significantly increased MSCs proliferation compared to the bare microcarriers. Further, immobilization of BMP-2 on microcarriers resulted in superior MSCs proliferation. Cell proliferation assay and immunofluorescent imaging results over two weeks converge to indicate the efficacy of PDA functionalization and immobilization of BMP-2 to boost cell expansion.

During the normal bone healing and regeneration process, while VEGF is predominantly expressed throughout the initial phases in order to induce blood vessel formation and re-establish vascularization, BMPs are uninterruptedly expressed to induce bone formation and remodeling. It has been found VEGF expression is upregulated in the first 10 days, with a peak around days 5 to 10^78–80^.

Cell-based therapy approaches for bone regeneration require delivery of the cells through a carrier, which guides the cell fate and induces both osteogenesis and vascularization^40,64^. The rationale behind the design of microcarriers in current study was intended to fulfill sequential delivery of VEGF and BMP-2. The amount of bone formation in vivo relies on duration of BMP-2 delivery to MSCs in the first four weeks, whereas the extent of vascularization relies on VEGF delivery to endothelial progenitor cells within the first 14 days^81,82^.

**Figure 4:**
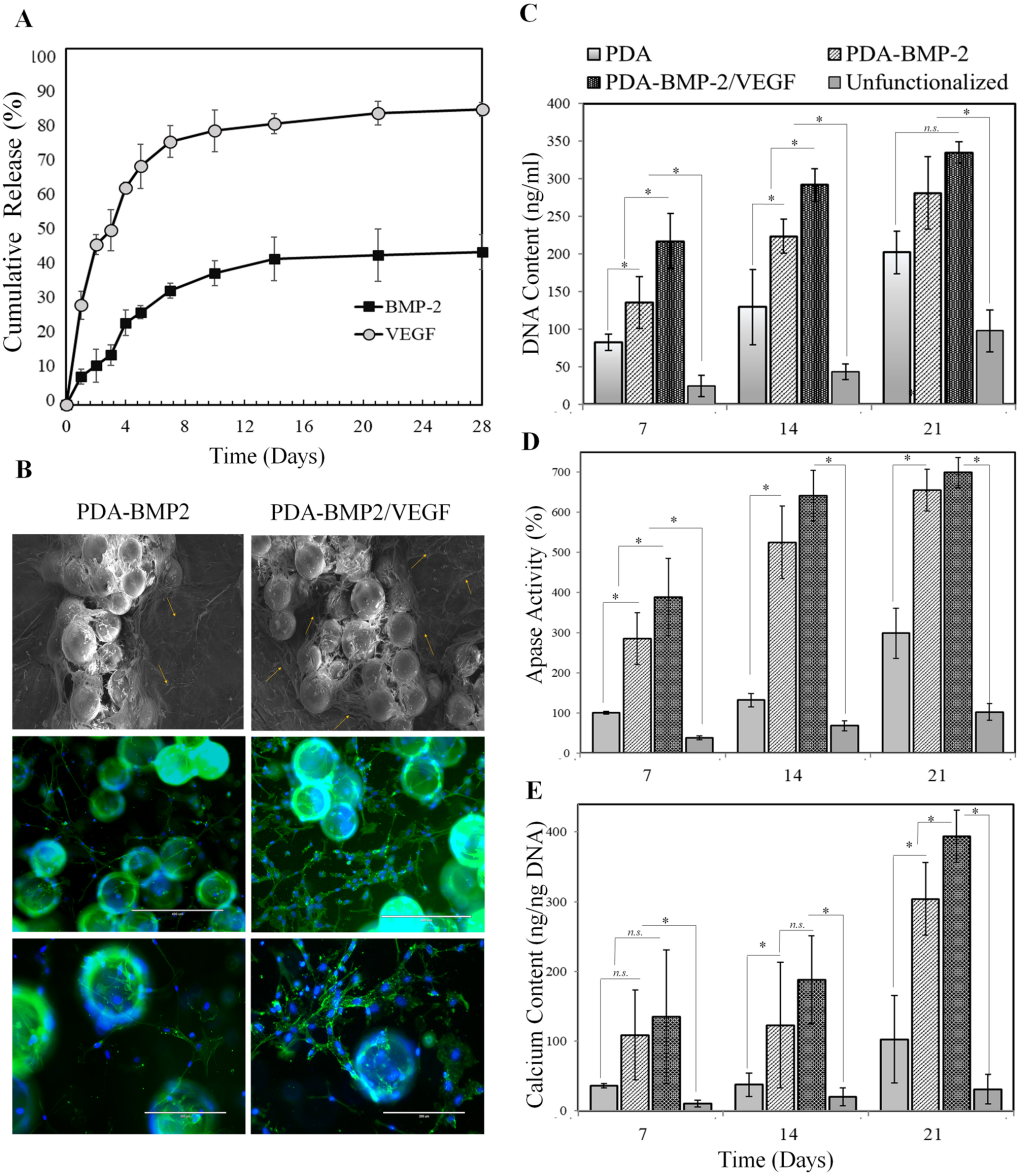
(**A**) The release pattern of VEGF and BMP-2 over PDA functionalized microcarriers in 4 weeks. (**B**) The scanning electron microscopy (SEM) and immunofluorescent images of tunneling formations of HUVECS in alginate RGD matrix containing PDA-BMP-2 microcarriers with or without VEGF encapsulation. (**C**) DNA content, (**D**) ALP activity, and (**E**) calcium content of the matrix with the co-delivery of BMP-2 and VEGF compared with the control groups. (*P < 0.05).

While VEGF encapsulation into the microcarriers provides sustained release of VEGF up to 4 weeks, immobilization of BMP-2 on the surface of the microcarriers provides bioavailability of BMP-2 for an extended period. As can be seen in the overlapped in vitro release profiles of VEGF and BMP-2 shown in Fig. 4A, while more that 80% of VEGF was released in 4 weeks, BMP-2 has only reached half its cumulative release.

Figure 4B–D demonstrates the injectable alginate-RGD hydrogel formulation supports the proliferation and differentiation of cell-seeded microcarriers along with pre-vascularization of the construct. The VEGF released from the cultured PLGA microcarriers entrapped into the hydrogel matrix showed a favorable outcome to enhance tunnel formation of HUVECs (Fig. 4B). It should be noted that covalent conjugation of adhesive extracellular matrix and cell surface proteins such as RGD into the alginate hydrogel have improved the cellular activity^60^. Alkaline phosphatase (ALP) has a significant role in cell differentiation and bone mineralization; thus, the ALP activity as an early osteoblastic differentiation marker was investigated to analyze the osteogenic differentiation of MSCs and determine bioactivity of the matrices. As seen in Fig. 4D, ALP activity in response to PDA coating was significantly higher than that of unfunctionalized microcarriers (*p* < 0.05). The effect of PDA coating to induce osteogenic differentiation and increase ALP activity has been reported in other studies^62,83,84^. An increase in ALP activity was observed for the PDA-BMP-2 and PDA-BMP-2/VEGF groups over 3 weeks, significantly higher than the PDA and unfunctionalized groups as the control groups (*p* < 0.05). Such observation indicates the osteogenic differentiation ability is elevated with co-delivery of growth factors, and suggests dual release of BMP-2 and VEGF improve osteogenic differentiation for 21 days. The effect of VEGF on osteogenic pathways is known, which results in BMP-2 upregulation and plays an important role in bone regeneration.

The capability of MSCs to mineralize inorganic phosphates during differentiation is one of the main evidences for the osteogenic differentiation. Figure 4E shows Calcium content for all groups gradually increased with incubation time. It should be noted that PDA coating resulted in higher calcium content compared to the unfunctionalized microcarriers. Catechol moieties on PDA coated microcarriers concentrate Ca^2+^ ions and induce nucleation of hydroxyapatite (HA) crystals. In fact, catechol moieties on the microcarriers surface increase the calcium precipitation, and local supersaturation of Ca^2+^ leads to the formation of HA crystals on the substrate^85,86^. On the other hand, PDA coating not only improves hydrophilicity of polymeric surfaces, but also provide protein conjugation through Michael addition onto the surface, which promotes cells adhesion. Actually, modulation of microcarriers with PDA functionalization have improved signal transduction of cell adhesion, proliferation and differentiation. It has been reported that the FAK/P38 and mitogen-activated protein kinases signaling pathways of MSCs are upregulated in response to PDA coating. These pathways are involved in proliferation and osteogenic differentiation of MSCs^85–87^.

Microcarriers comprising PDA-BMP-2 and PDA-BMP-2/VEGF were found to show superior calcium content, which implies co-delivery of growth factors induce improved osteoinduction stronger than PDA coating alone. There are several studies presenting strong osteoinductivity of BMP-2 is mainly attributable to its induced mineralization pathways in MSCs. It has been reported the mineral density within de novo bone can be increased by 33% in scaffolds containing both BMP-2 and VEGF, as compared to those containing only BMP-2^48^.

**Figure 5:**
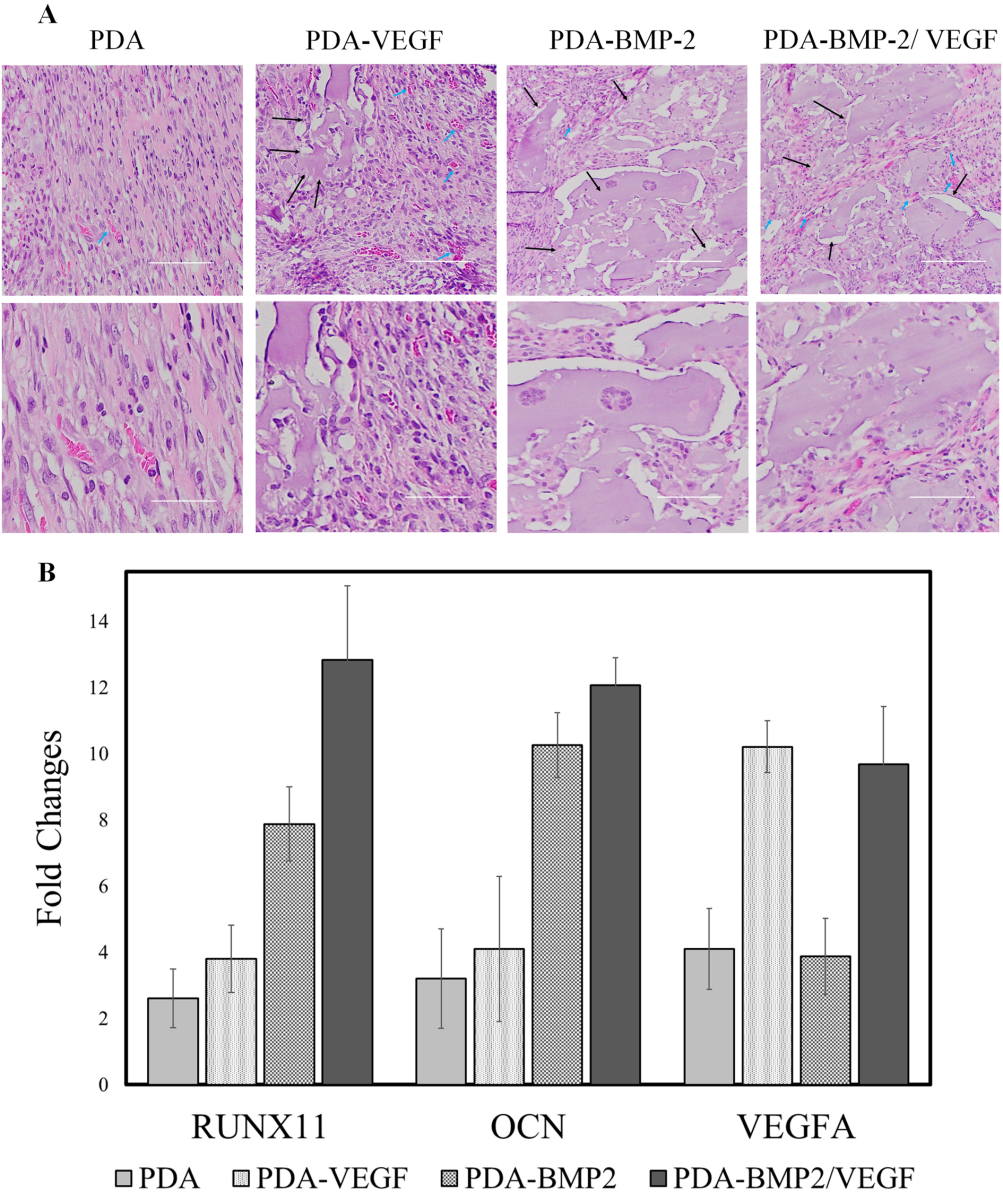
(**A**) Hematoxylin and eosin staining of sections from decalcified gel matrices loaded with cell-laden microcarriers: PDA-treated (as control group), VEGF-encapsulated, BMP‐2 conjugated, and VEGF-encapsulated microcarriers conjugated with BMP-2 after 6 weeks subcutaneous implantation. (**B**) Real-time PCR analysis for RUNX2, OCN and ALP after 6 weeks subcutaneous implantation.

In order to study capability of the designed cell-laden constructs for bone formation in vivo, hydrogels incorporated with the four microcarrier groups of PDA-treated (as control group), VEGF-encapsulated, BMP‐2 conjugated, and VEGF-encapsulated microcarriers conjugated with BMP-2, were subcutaneously implanted into rat animal models. The histological evaluation of tissue after 6 weeks showed uneven wound healing for all groups, and no sign of severe inflammation or foreign body reaction was observed (Fig. 5). The control group did not show any evidence of bone formation, and only connective tissue ingrowth was found. Various extent of bone tissue formation, including thin mineralized tissue and large marrow spaces with connective tissue cells and occasional hematopoietic cells, was observed for the VEGF, BMP-2, and BMP-2/VEGF groups. The osteoblast-like cells were found to be regenerating along with the mineralized tissue. Small regions of bone tissue surrounded by a connective tissue formed in the VEGF group, and blood vessels were found to be more evident compared to the control and BMP-2 groups. However, a superior bone tissue accompanied with pronounced blood vessels were found to form in the case of BMP-2/VEGF group in comparison with other groups (Fig. 5A).

The mRNA expression level of RUNX2, OCN, and VEGFA genes were characterized (Fig. 5B). The results of qPCR analysis are consistent with the histological assessment. The osteogenesis markers were found to be significantly more expressed for the BMP-2 group than the VEGF and control groups, while VEGFA was significantly lower expressed in BMP-2 group. The VEGF group showed a 3.8 and 4.1‐fold higher expression of RUNX2 and OCN, respectively, compared to the control group (Fig. 5B). Statistical analysis revealed a significant RUNX2 expression for the BMP-2/VEGF group (*p* < 0.001). However, the difference between BMP-2/VEGF and BMP-2 groups were less in OCN expression level. The BMP-2 and VEGF signaling pathways may enhance the RUNX2 mRNA level that stimulate the osterix gene expression^88,89^. However, this combination was found to be less effective in pathways related to OCN expression.

The beneficial outcome of BMP-2 and VEGF co-delivery on bone mineralization and bone protein expression can be explained by their synergistic effects. VEGF is a substantial mediator inducing angiogenesis while also playing a key role in osteogenesis. It has been confirmed VEGF expression and low oxygen level in bone defect upregulate BMP-2, which is essential in bone repair. VEGF was reported to significantly enhance BMP-2 expression in microvascular endothelial cells^90^.

Geuze et al*.*^91^ reported effect of BMP-2 and VEGF release timing on ectopic bone regeneration While they created sustained-release profiles using PLGA microparticles, fast-release profiles were achieved by rapidly degrading gelatin particles. The extent of orthopedic and ectopic bone regeneration was shown to be dependent on the release timing of BMP-2. However, their results did not support the effects of VEGF on the amount of bone formation, which can be related to the dose and duration of VEGF release. In another study, collagen matrices loaded with both VEGF and BMP-2, compared to delivery of BMP-2 alone, resulted in more capillary and bone formation in an ectopic site after 3 weeks. Such observation supports the synergistic effect of co-delivery of osteogenic and angiogenic factors to promote ectopic bone formation^92^.

## Conclusion

Cell-therapy in tissue regeneration have generated profound enthusiasm. Yet, outcomes of many trials have been unsatisfactory, which is due to the lack of proper vehicles for controlled cellular function and growth factors delivery. Although effect of VEGF and BMP-2 controlled co-delivery using different biomaterials on vascularization and osteogenesis has become evident, ideal delivery vehicles to replicate the natural release pattern of the growth factors still need to be explored. Herein, we demonstrated conjugation of BMP-2 via PDA chemistry onto monodisperse polymeric microcarriers encapsulating VEGF can replicate an optimal spatiotemporal bioavailability of the growth factors. The engineered microcarriers demonstrated a great potential in dynamic culture technique to extensively expand and guide MSCs fate toward clinical bone stem cell therapy. Besides showing superior bioactivity in vitro, transplanted MSCs-laden microcarriers through an injectable hydrogel matrix revealed to induce vascularization and ectopic bone formation in vivo.

A feasible process to scale-up the designed growth factor incorporated microcarriers is high-density microfluidic platform consisted of multiple microchannels. Additionally, it is worthy of note, although in this study we addressed bone regeneration, the need for vascularization plays a key role in development of the most tissue-engineered constructs. Thus, the outcome of this study is readily expandable for engineering of other tissues.
